# RESIDENT, STAFF, AND FACILITY FACTORS ASSOCIATED WITH AFFECT BALANCE

**DOI:** 10.1093/geroni/igz038.2061

**Published:** 2019-11-08

**Authors:** Ann M Kolanowski, Liza Behrens, Kimberly Van Haitsma, Barbara Resnick

**Affiliations:** 1 Penn State, University Park, Pa., United States; 2 Penn State, University Park, Pennsylvania, United States; 3 University of Maryland, University Park, Maryland, United States

## Abstract

Affect balance is a concept that is measured by comparing the relative frequency of experiencing positive affect versus negative affect. A higher ratio of positive to negative affect has been associated with greater well-being, improved function and resilience. Baseline data from an ongoing pragmatic trial were used to determine the resident, staff and facility factors associated with affect balance in 325 nursing home residents with moderate to severe cognitive impairments. Measures of resident demographics, cognitive status (BIMS), function (Barthel Index), staff hours, staff knowledge of person-centered care approaches, facility policies and the facility environment were taken. Affect balance was measured using items from the Quality of Life in AD scale. Initial correlational analyses indicate that affect balance is associated with being male (p=0.03), having better function (P=0.04) and receiving a greater number of RN and CNA hours of care (p=0.003 and P=0.02 respectively).

